# Supplementary material for: Complex three-dimensional self-assembly in proxies for atmospheric aerosols
Source: Nat Commun. 2017 Nov 23;8:1724. doi: 10.1038/s41467-017-01918-1 (PMC5701067; doi:10.1038/s41467-017-01918-1)
Supplement: Supplementary file 1 — Supplementary Information [file 41467_2017_1918_MOESM1_ESM.pdf]

Supplementary Information:

Supplementary Note 1: Confirmation of Complex 3D Self-Assembly and Assignment of Lyotropic Phases

This section shows SAXS patterns from different lyotropic phases we observed in sodium oleate/oleic acid/brine droplets prior to ozonolysis, with peak position analysis confirming the identities of the less common inverse micellar Fd3m (cubic close-packed) and $P6_3/mmc$ (hexagonal close-packed) phases.

Supplementary Figure 1(a–e) shows radial profile SAXS patterns from (a) the $P6_3/mmc$ phase shown in the main text; (b) the same $P6_3/mmc$ phase observed in a subsequent experiment on another droplet; (c) an Fd3m cubic close-packed inverse micellar phase; (d) an inverse micellar phase; and (e) a lamellar phase. Confirmation of the identities of the $P6_3/mmc$ and Fd3m phases is shown in Supplementary Figure 1(f) and (g), respectively.

Predicted values of
$m = \sqrt{(h^2 + k^2 + l^2)}$ for the Fd3m phase,[1] and
$m = \sqrt{\frac{4}{3}(h^2 + k^2 + hk) + \frac{l^2}{R^2}}$ for the $P6_3/mmc$ phase[2] are calculated for each symmetry-allowed reflection from a set of planes defined by Miller indices hkl; R is the ratio of unit cell dimensions c/a, which has a theoretical value of 1.633 for a 3D hexagonal close-packing of spheres of spacegroup[2] $P6_3/mmc$. These calculated values of m are plotted against the experimentally observed peak positions 1/d. If the assignment is correct, the plot should be linear with slope equal to unit cell dimension a (labelled in the inset schematic figures in Supplementary Figure 1 (f), (g)), and pass through the origin.[1] For the $P6_3/mmc$ phase, the optimized data was fit using a value of R = 1.631, and the proportionality demonstrated by the m *vs.* 1/d plots (Supplementary Figure 1 (f), (g)) confirm the phase assignment. The slopes of the plots give unit cell dimension values of a = 76 and 113 Å respectively for the $P6_3/mmc$ and Fd3m phases. In the former case the unit cell dimension a is equal to the centre-to-centre distance of adjacent micelles, and therefore the effective micelle diameter assuming close-packed spheres. In the latter case a is equal to $\sqrt{2} \times$ diameter. From this information we can estimate micelle diameter values from the $P6_3/mmc$ and Fd3m phases of 76 and 80 Å, respectively.

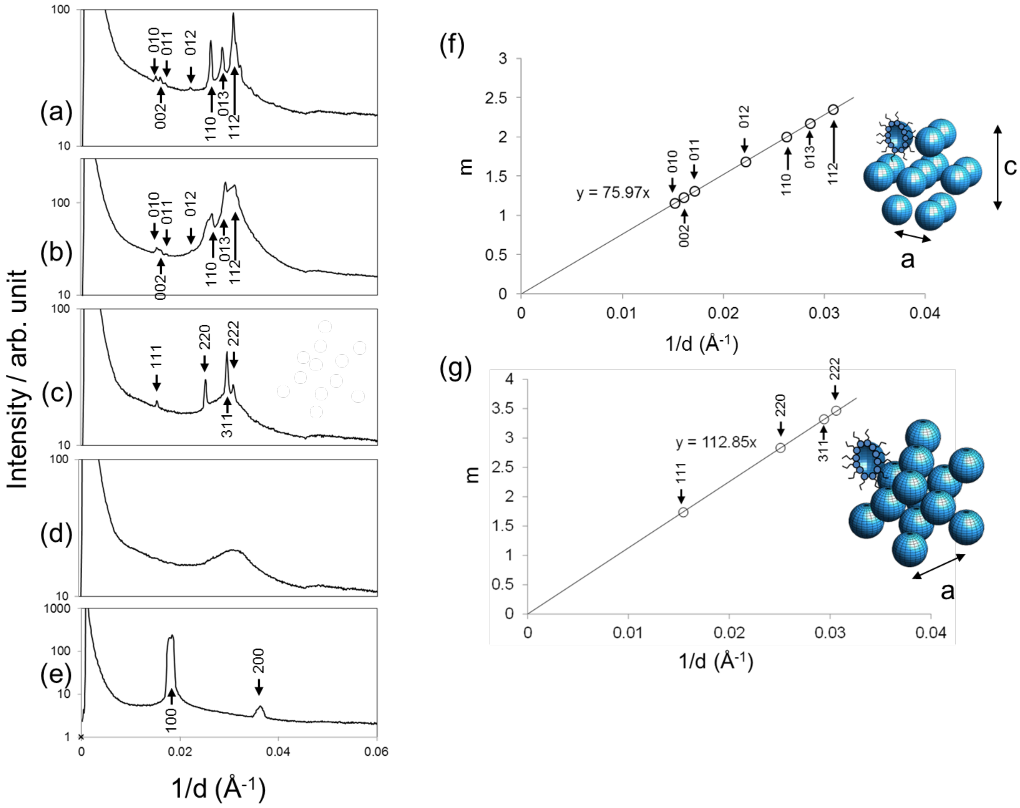

Supplementary Figure 1: Assignment of complex 3D self-assembled phases: SAXS patterns from (a) the P6$_3$/mmc phase shown in the main text (88% RH) approx. 3000 s after injection; (b) the same P6$_3$/mmc phase observed in a subsequent experiment on another droplet at 98% RH approx. 2400 s after injection; (c) an Fd3m cubic close-packed inverse micellar phase at 97% RH approx. 2000 s after injection; (d) an inverse micellar phase from the same drop as (c) at 97% RH after a further approx. 600 s; and (e) a lamellar phase approx. 7200 s after injection at 76% RH followed by dehydration to 64% RH then increase in relative humidity to 97% RH. (f) and (g) illustrate the phase assignment (see text for details).

Supplementary Note 2: Additional SAXS Data for Ozonolysis Experiments

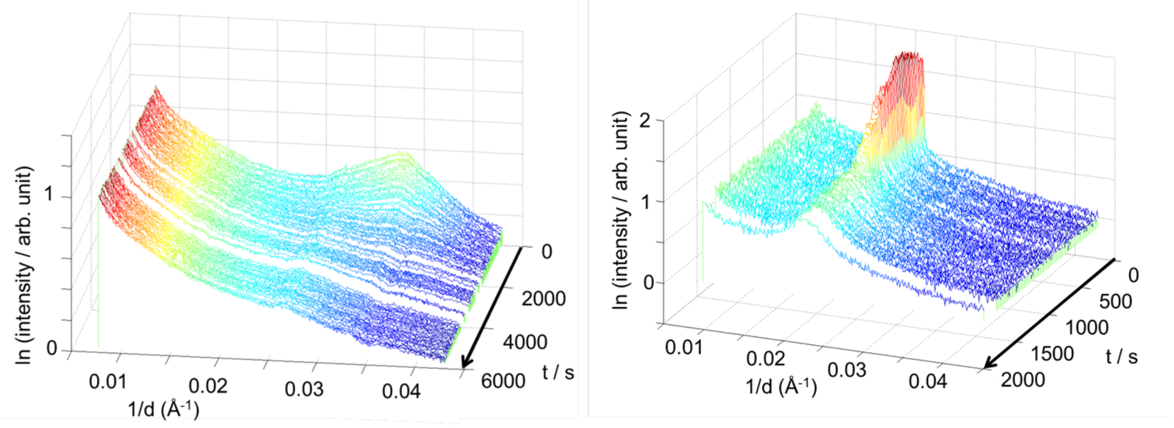

Supplementary Figure 2: Additional SAXS data obtained from ozonolysis experiments: SAXS data showing droplets initially in micellar (left) and lamellar (right) phase, changing phase during exposure to ozone.

Supplementary Note 3: Complementary Data on Ozonolysis Experiment Reported in Main Manuscript

This section complements the data presented in the main manuscript. Supplementary Figure 3(a) contrasts the Raman spectra obtained during (t = 1577 s) and after exposure to ozone (t = 5220 s); a characteristic change is the loss of the C=C peak at ~ 1650 cm^{-1}; formation of nonanoic acid in the droplet was confirmed: the final Raman spectrum at t = 5220 s shows in addition to the absence of the C=C band a small but characteristic change in CH band shape and the loss of a small peak at ~ 3020 cm^{-1}.

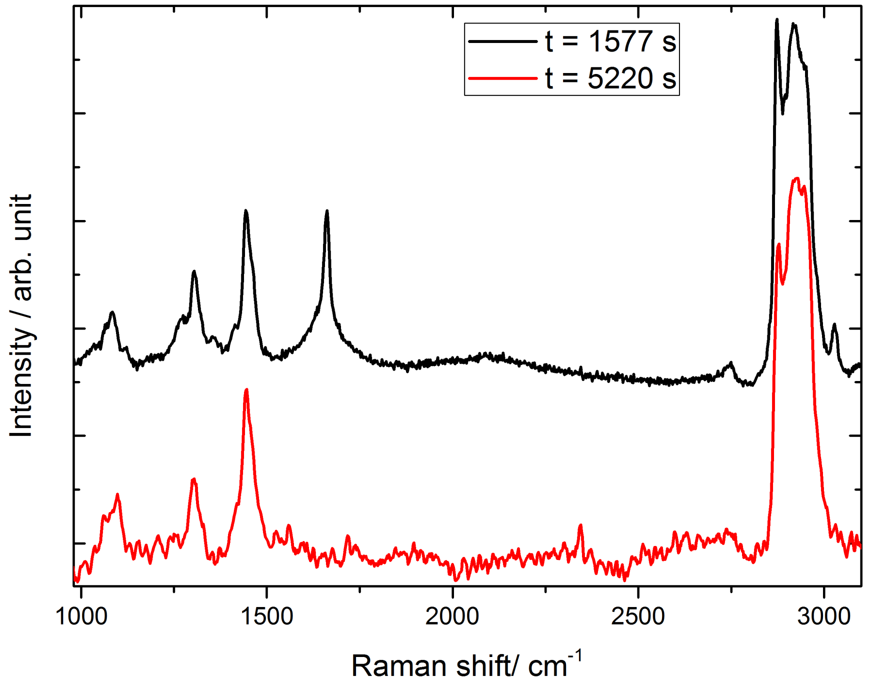

Supplementary Figure 3(a): Ozonolysis experiment: Raman spectra illustrating formation of nonanoic acid in the levitated droplet: loss of C=C peak (at ~ 1650 cm^{-1}), a characteristic change in CH band shape and disappearance of a small peak at ~ 3020 cm^{-1} during ozonolysis (same experiment as displayed in Fig. 4 in the main manuscript).

Supplementary Figure 3(b) illustrates the changes in water content observed during ozonolysis. Following loss of the water peak at peak (~ 3070–3700 cm^{-1}; spectra are normalized to the CH_2 deformation band at ~ 1442 cm^{-1} as Fig. 4(b) in the main manuscript). Initial water uptake is followed by loss in water until stabilization at ca. t = 2000 s. This coincides with the loss of the complex self-assembly of the aerosol proxy.

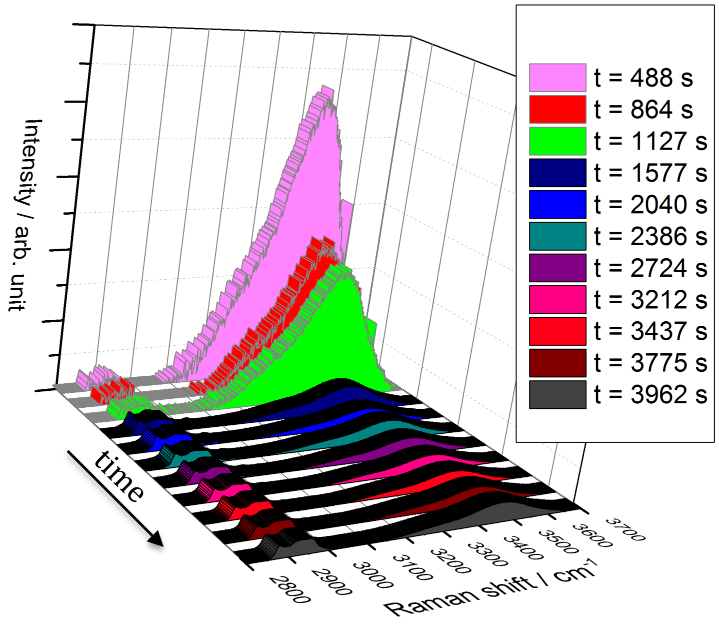

Supplementary Figure 3(b): Ozonolysis experiment: Raman spectra illustrating initial uptake of water (at ~ 3070–3700 cm^{-1}) during ozonolysis (following initial dehydration illustrated in Fig. 3(b) in main manuscript) and subsequent reduced level of water content (same experiment as displayed in Fig. 4 in the main manuscript; spectra at t = 488–1127 s are quite noisy hence not included in Fig. 4 for visual clarity; spectra are normalized to the CH_2 deformation band at ~ 1442 cm^{-1} that scales well with the displayed CH band at ~ 2850–3000 cm^{-1}).

2D versions of the 3D figures displayed as Figs. 3(b) and 4(b) in the main manuscript are inserted below as Supplementary Figure 3(c) and 3(d), respectively.

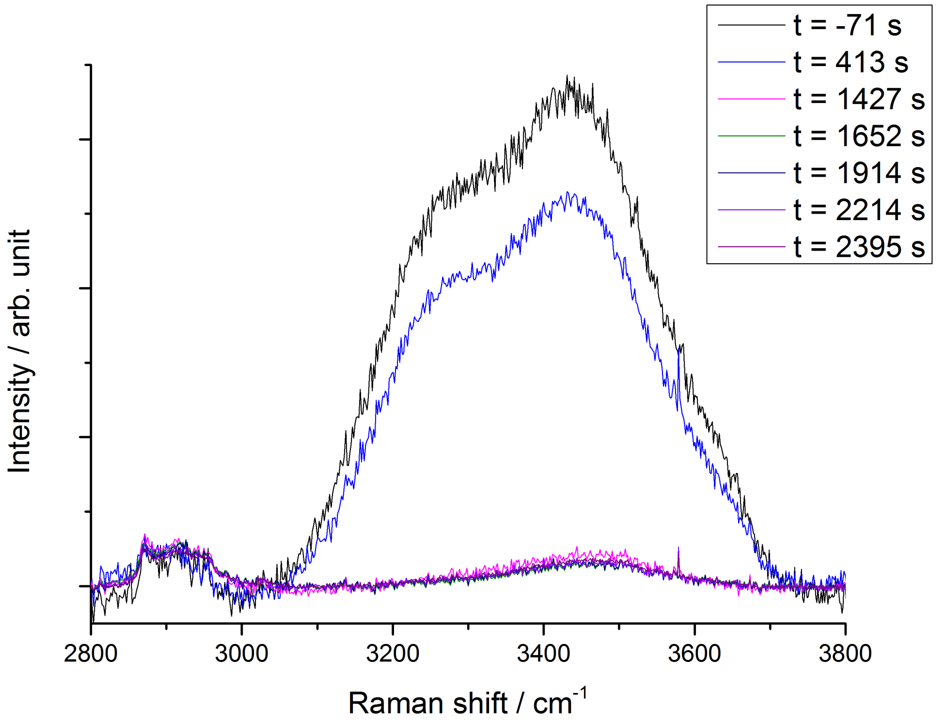

Supplementary Figure 3(c): Dehumidification experiment (displayed as 3D figure in Fig. 3(b) in the main manuscript): Raman spectra illustrate the reduction of the broad H_2O peak (~ 3070–3700 cm^{-1}; spectra are normalized to CH band at ~ 2850–3000 cm^{-1}).

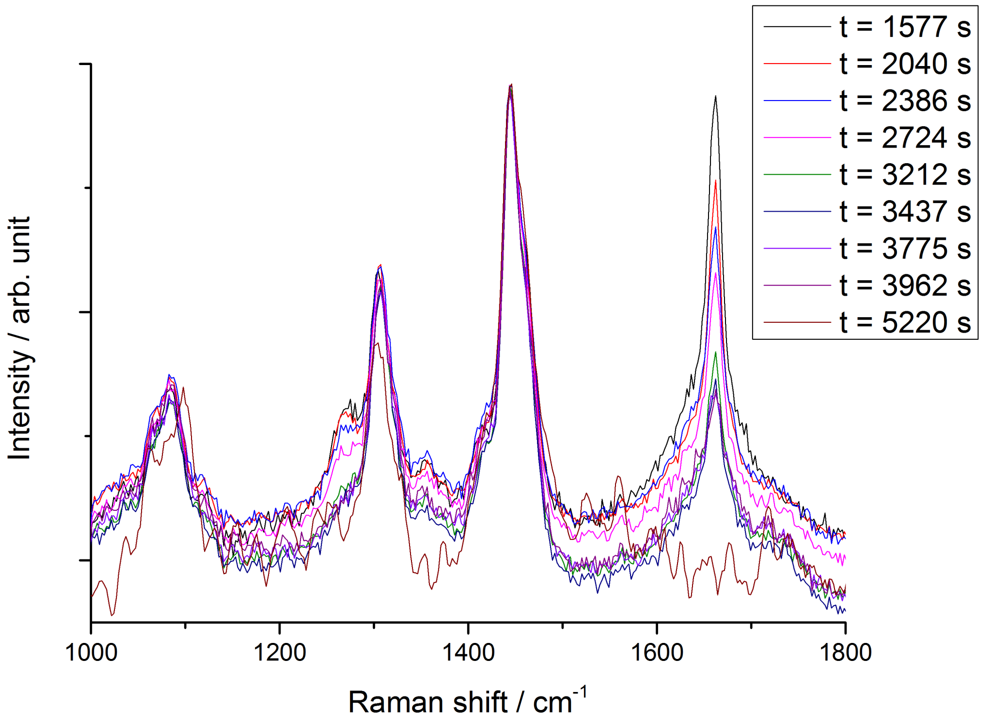

Supplementary Figure 3(d): Ozonolysis experiment (displayed as 3D figure in Fig. 4(b) in the main manuscript): Raman spectra illustrating the clear reduction of the C=C peak at ~ 1650 cm^{-1} (spectra are normalized to the CH_2 deformation band at ~ 1442 cm^{-1}).

Supplementary References

1. Seddon, J.M. et al. Inverse cubic liquid-crystalline phases of phospholipids and related lyotropic systems. *Journal of Physics Condensed Matter*, **1990**, *2*, 285–290
2. Clerc, M., A new symmetry for the packing of amphiphilic direct micelles. *Journal de Physique* II, **1996,** *6*, 961–968
